# Experimental Demonstration of a Spin Logic Device with Deterministic and Stochastic Mode of Operation

**DOI:** 10.1038/s41598-018-29601-5

**Published:** 2018-07-30

**Authors:** Punyashloka Debashis, Zhihong Chen

**Affiliations:** 1 School of Electrical and Computer Engineering, Purdue University, West Lafayette, IN 47907 USA; 2 Birck Nanotechnology Center, Purdue University, West Lafayette, IN 47907 USA

## Abstract

Spin based logic devices have attracted a lot of research interest due to their potential low-power operation, non-volatility and possibility to enable new computing applications. Here we present an experimental demonstration of a novel spin logic device working at room temperature without the requirement of an external magnetic field. Our device is based on a pair of coupled in-plane magnetic anisotropy (IMA) magnet and a perpendicular magnetic anisotropy (PMA) magnet. The information written in the state of the IMA magnet is transferred to the state of the PMA magnet by means of a symmetry breaking dipolar field, while the two layers are electrically isolated. In addition to having the basic tenets of a logic device, our device has inbuilt memory, taking advantage of the non-volatility of nanomagnets. In another mode of operation, the same device is shown to have the functionality of a true random number generator (TRNG). The combination of logic functionality, nonvolatility and capability to generate true random numbers all in the same spin logic device, makes it uniquely suitable as a hardware for many new computing ideas.

## Introduction

**Figure 1:**
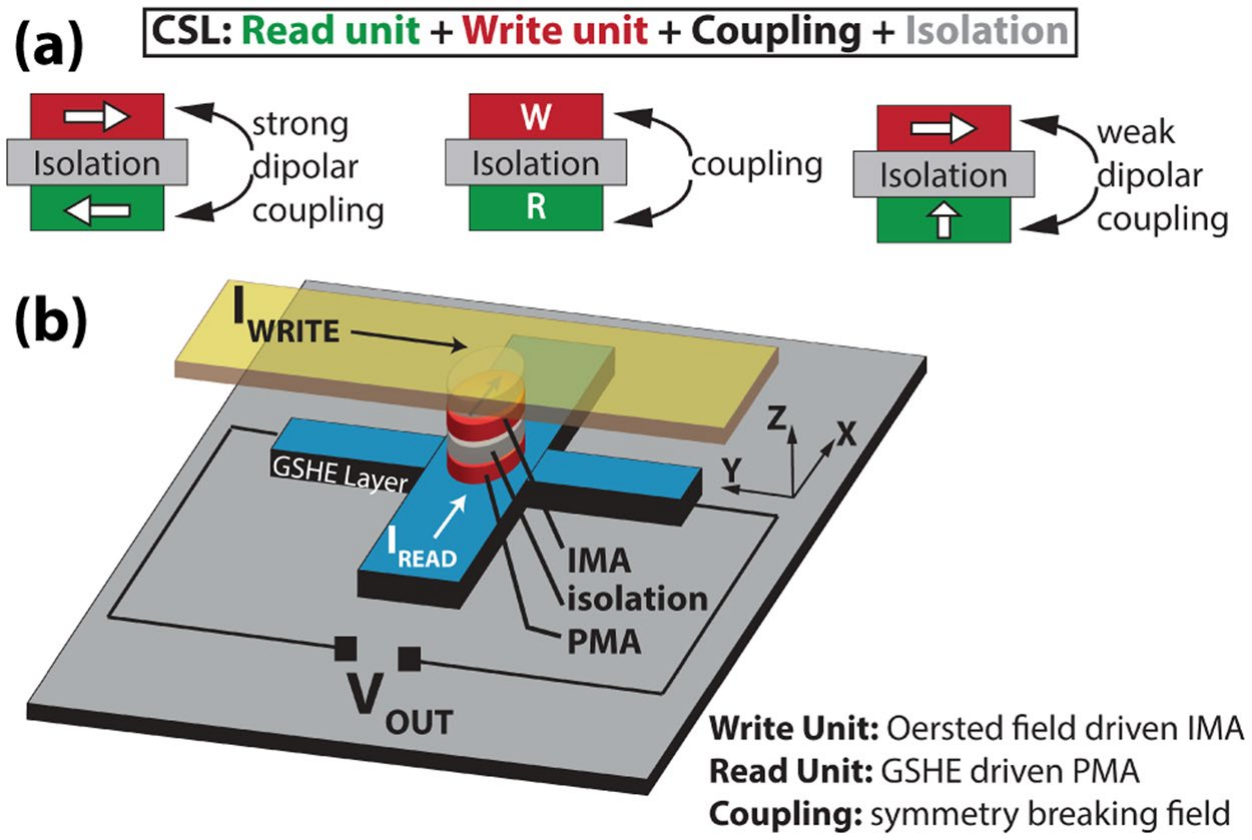
(**a**) Center: Charge-coupled Spin Logic device containing READ and WRITE units that are electrically separated but magnetically coupled. Left: The original proposal was based on two IMA magnets and a strong coupling between them that can switch them in unison. Right: Our idea is to have an IMA magnet and a PMA magnet. A much smaller dipolar field from the IMA magnet is required to break the symmetry of GSHE switching of the PMA magnet underneath, achieving information flow from WRITE to READ unit. (**b**) Schematic of the coupled IMA-PMA device.

Building logic units with spintronic elements is a topic of great interest as they can offer the functionality of a logic device at lower power and at the same time serve as memory elements, owing to the non-volatile nature of nanomagnets. Driven by this idea, many proposals of spin based logic devices have been widely discussed^1–4^. Charge-coupled spin logic (CSL)^2^ is one of the most promising implementations due to the use of robust charge currents for long transport distances as terminal quantities. The CSL design consists of a WRITE unit into which information can be written by an external stimulus and a READ unit from which the information can be read out and supplied to the next stage as input. Most importantly, the WRITE and the READ units must be electrically isolated to eliminate feedback, at the same time should be directionally coupled so that information can be transferred from the WRITE unit to the READ unit (Fig. 1(a) center).

In the original CSL proposal, giant spin Hall effect (GSHE) was proposed to be the writing mechanism and magnetic tunnel junction (MTJ) was suggested as the READ unit. Although the physics and operation of GSHE and MTJ are both well established, a final experimental demonstration of a CSL like spin logic device that meets design criteria of a logic device is still missing. One of the essential missing components is the experimental demonstration of an electrically isolated but directionally coupled READ and WRITE unit. In this work, we present a new implementation that demonstrates this central aspect of the CSL device, while utilizing simple mechanisms to implement an electrical READ and WRITE unit. The presented device has (i) an IMA magnet driven by Oersted field as the WRITE unit, (ii) a PMA magnet switched by the GSHE of Ta and utilizing anomalous Hall effect (AHE) as the READ unit and (iii) the symmetry breaking field from IMA to PMA as the directional coupling (Fig. 1(b)). The nearby IMA magnet dictates the response of PMA to the GSHE spin torque. By writing information into the IMA through Oersted field and reading information from the AHE of the PMA, a new CSL device is successfully implemented with the combined PMA-IMA stack.

In contrast to the originally proposed implementation of CSL (Fig. 1(a) left), the coupling between WRITE and READ is realized by a very small dipolar field from the IMA that is sufficient to break the symmetry of GSHE switching of the PMA (Fig. 1(a) right). This weak coupling relaxes the design constraints on the isolation layers and magnet dimensions^5^, i.e., we can now design thicker isolation layers for improved read-write isolation and endurance, and thinner magnets for lower operation energy. Moreover, using the CoFeB/MgO stack with interfacial PMA in our device design enables possibility for novel voltage modulation of magnetic properties to achieve ultra-low power operation^6–10^.

We emphasize that the read and write mechanisms of the presented device can be replaced by more efficient technologies. For example, the WRITE unit can employ spin orbit torque (SOT) switching of the IMA magnet by having a GSHE material like Tantalum adjacent to it^11^. It can also utilize recent advances in voltage control of magnetization^12,13^. The READ unit can include a PMA-MTJ^14^ similar to the original CSL proposal. These mechanisms can be integrated based on the application needs or desired performance metrics as long as they fulfill the requirements of the coupled IMA-PMA device design shown in Fig. 1(c). The specific design presented in this paper is just one of the designs used for easy integration, while the main focus is to experimentally demonstrate a CSL operation as the first proof-of-concept spin logic unit with input/output isolation and directed coupling. Finally, we demonstrate that the same device is capable of producing true random binary digits through the stochastic switching of its PMA, when the read current through the GSHE layer is above a certain value. We show that this functionality is only possible if the amount of dipolar field exerted by the IMA on the PMA is engineered properly.

## Experimental Approach and Results

**Figure 2:**
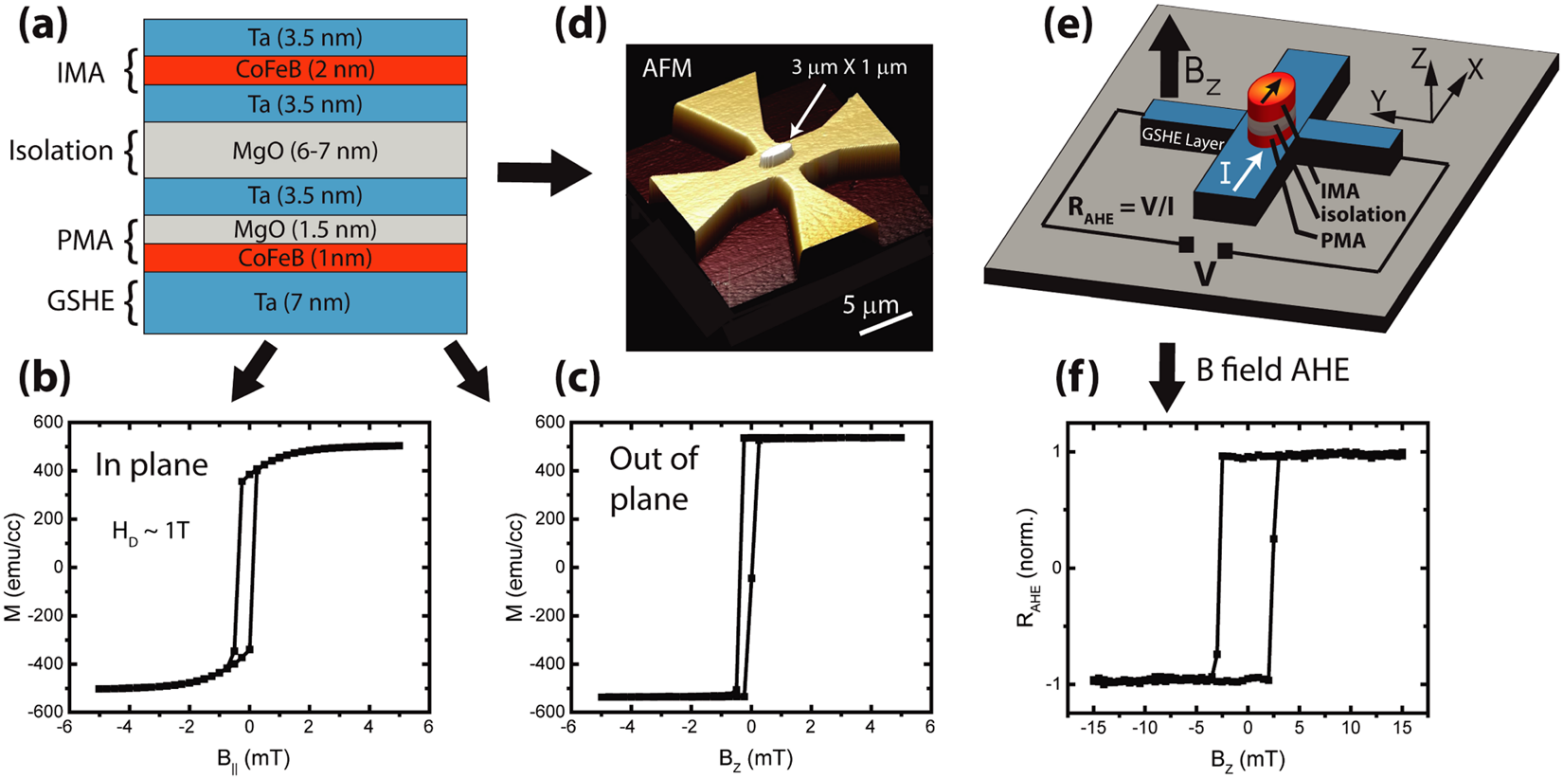
(**a**) The composite stack of IMA and PMA magnets, separated by 6–7 nm MgO as the electrical isolation. The stack sits on 7 nm Ta that serves as the GSHE layer for the PMA magnet switching. (**b**,**c**) SQUID characterization of separately deposited IMA and PMA magnetic stack, showing good in-plane and out-of-plane ferromagnetic behavior, respectively. (**d**,**e**) AFM and schematic of the fabricated device having a Ta Hall cross and an elliptical island of the composite IMA-PMA stack. (**f**) AHE resistance vs. externally applied out-of-plane magnetic field, showing abrupt hysteretic behavior that is indicative of the PMA in the etched island.

We begin with depositing a composite stack of PMA and IMA magnet separated by 6–7 nm MgO that serves as the electrical isolation between them. This composite stack sits on a 7 nm Ta GSHE layer (Fig. 2(a)). Vibrating sample magnetometry (VSM) measurements using a superconducting quantum interference device (SQUID) on control samples reveal good ferromagnetic behaviors, with a high ratio of remnant moment to saturation moment and abrupt magnetization reversal for both IMA and PMA layers (Fig. 2(b,c)). Next, the Ta layer is patterned into a Hall bar with the composite IMA-PMA stack sitting on top shaped into an ellipse with major and minor diameter of 3 μm and 1 μm, respectively (Fig. 2(d)). AHE measurements done with external out-of-plane field (Fig. 2(e)) show square hysteresis loop (Fig. 2(f)), confirming two distinguished magnetization states. The IMA signal does not impact this measurement as it is isolated by the MgO layer. Note, the coercive field in Fig. 2(f) is larger than that in Fig. 2(c), as a result of patterning the PMA stack into a smaller island^14,15^. The impact of the stray magnetic field from the IMA on the PMA magnet’s coercive field is minimal as both of its in plane and out of plane components are less than 1 mT.

### Switching PMA with in plane polarized spin current

As first shown by Miron *et al*.^16^ and Liu *et al*.^17^, in-plane polarized spin currents can produce SOT that can efficiently rotate the magnetization of a PMA deterministically in the presence of a small symmetry breaking in-plane field. In this scenario, the critical current needed to switch a PMA magnet can be much smaller than for an IMA with the same energy barrier, due to the absence of a demagnetization field for PMA (see Supplementary Information, Section S10). However, the requirement of an in plane field to deterministically switch the PMA magnetization from “up” to “down” (or “down” to “up”) makes it undesirable for computing applications. Many approaches have been demonstrated to get rid of this external field. These approaches introduce a built-in symmetry breaking field, by means of either a tilted anisotropy^18^, lateral structural anisotropy^19^, interlayer exchange coupling^20^ or the GSHE of an antiferromagnet^21^. All these methods rely on a mechanism that produces a fixed in-plane symmetry-breaking field that cannot be manipulated easily once the device is fabricated.

### Field free switching by means of a coupled IMA

We eliminate the requirement of the external symmetry-breaking field by using the dipolar field of the IMA magnet placed on top of the PMA magnet. This approach eliminates the challenging fabrication requirements of refs^18,19^ and does not require complicated interlayer and antiferromagnetic material stack as in the case of refs^20,21^. More importantly, this approach allows for an independent electrical control of the magnetization of the IMA magnet, enabling an input and an output control for logic functionality as will be described in later sections.

**Figure 3:**
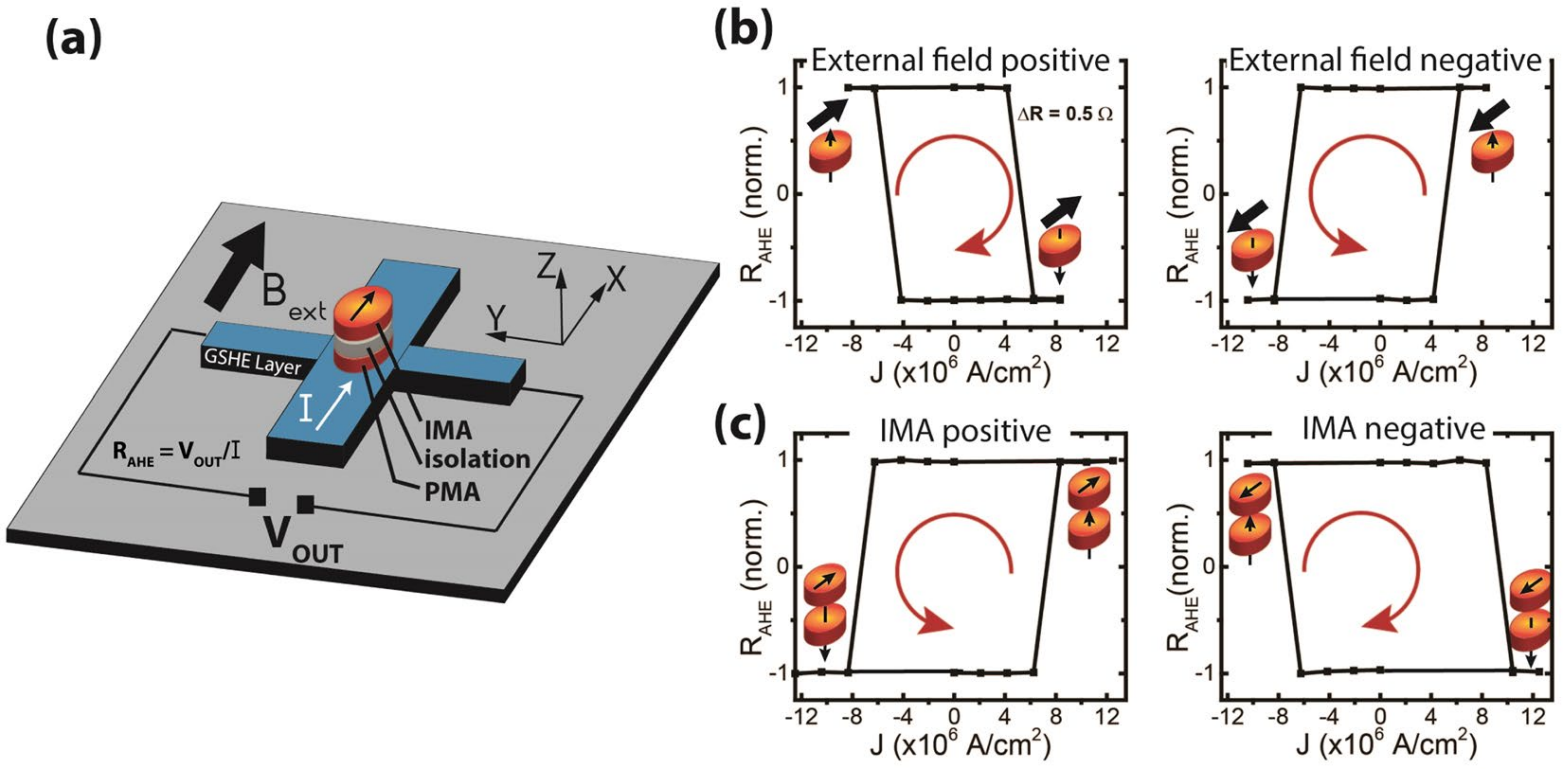
(**a**) Device schematic with measurement configuration (**b**) GSHE switching loops in the presence of an external symmetry-breaking field. For positive magnetic field (+100 mT), positive current switches the PMA magnet from “up” to “down” magnetization, while negative current switches the PMA magnet from “down” to “up” magnetization, giving a clockwise loop direction. This is reversed when the external field is negative (−100 mT), showing a counter clockwise switching loop. (**c**) GSHE switching loops are obtained without the aid of an external field. The loop directions are opposite to that of the external field case, indicating that a dipolar field from the IMA magnet serves as the symmetry breaking field for the GSHE switching of the PMA magnet.

The measurement schematic is shown in Fig. 3(a). First, we carry out switching loop measurements of the PMA magnet with the assistance of an external magnetic field of +/−100 mT, as shown in Fig. 3(b). The polarity of the external field and applied current direction determine the final magnetic state of the PMA as “up” or “down”, represented by the two R_AHE_ levels. It should be noted here, that the effect of the IMA dipolar field, which is expected to be no more than 1 mT, is overshadowed by the large external in plane field. The switching loop direction and symmetry is consistent with that observed by Liu *et al*.^17^ For SOT switching of PMA magnets without any assistance from heat induced thermal activation, the analytical expression for critical switching current is given by^17^:

1 $${J}_{sw,PMA}\approx \frac{2e}{\hslash }(\frac{1}{2}{M}_{S}{H}_{C}{t}_{PMA})(\frac{1}{{\theta }_{SH}})(\frac{1}{1-sech({t}_{GSHE}/{\lambda }_{SF})})$$

where M_S_ and H_C_ are the saturation magnetization and the switching field of the PMA magnet, t_GSHE_ and t_PMA_ are the thickness of the GSHE and PMA magnet layer, θ_SH_ is the spin Hall angle and λ_SF_ is the spin diffusion length of the GSHE layer. Substituting numbers measured from the experiment and taking λ_SF_ = 1.4 nm and θ_SH_ = 0.07 from ref.^17^, J_SW,PMA_ = 3.6 × 10^6^ A/cm^2^ is calculated. Although the above expression for critical current is for a monodomain magnetic body with H_C_ ≈ H_K_, and is not expected to match our experiment with micron sized magnets, the calculated number is consistent with the experimental values in Fig. 3. We also performed measurements for switching phase plots of another similar device (details in Supplementary Information, Section S2) to establish that the switching mechanism is same as that of Liu *et al*.^17^.

Next, we carry out the switching loop measurements in the absence of external magnetic field (Fig. 3(c)). The IMA magnet on top exerts a fringing dipolar field on the PMA magnet, which is strong enough to break the symmetry of the SOT switching. In the case where the IMA magnet is initialized along the positive (+x) direction, the PMA magnet senses its dipolar field pointing towards the negative (−x) direction and switches along the anti-clockwise loop as if it were sitting in a negative external field. Similarly, IMA magnet initialized along the negative direction produces a clockwise switching loop, consistent with a positive externally applied field. Based on calculations taking into account the IMA magnet size, saturation magnetization and separation from the PMA magnet, the average in plane dipolar field sensed by the PMA magnet is predicted to be ~0.7 mT (Supplementary Information, Section S8), which is strong enough to induce deterministic switching of the PMA. It is important to note that the magnetic field coming out of the IMA magnet contains both in plane and out of plane components, especially near the edges, as the two magnets are lithographically defined to have ~100% overlap. Nevertheless, the PMA magnet switching loop still reflects the same symmetries as in the case of the uniform external in plane field. Also, the fact that the PMA magnet switches between two distinct states with the same ΔR amplitude, implies that it behaves as a “whole” and does not break into domains, even in the presence of such a non-uniform field. We further confirm that the field free switching we observe is not due to domain wall propagation as seen by Li *et al*.^22^ Unlike in their experiment, we observe that the direction of our PMA switching loop is independent of its initial magnetization direction (see Supplementary Information, Section S4).

During preparation of the manuscript, the authors noticed that a similar approach for field free switching has recently been reported by Zhao *et al*.^23^ Nevertheless, our approach provides unique aspects that are not included in ref.^23^: First, we show complete electrical control of the device by adding a gold bar on top that switches the IMA magnet by means of Oersted field (section II C). Secondly, different from their structure, the thick MgO layer in our structure ensures that the isolation remains intact while the current pulsing of the PMA magnet takes place through many read-write cycles of the device (details of MgO leakage characterization is provided in the Supplementary Information, Section S6). Lastly, this thicker MgO allows us to tune the dipolar coupling in such a way that we can uncover a new stochastic mode of operation (section II D), which has not been experimentally observed elsewhere in symmetry broken composite GSHE/PMA structures. Details of the guidelines for choosing the isolation layer to enable stochastic operation at high read currents while obtaining deterministic switching at low currents is presented in the Supplementary Information, Section S8.

Here, we have eliminated the requirement of an external symmetry breaking field and successfully switched a PMA magnet by introducing an electrically isolated IMA magnet layer. However, initializing the IMA magnet still requires an external magnetic field, which needs to be eliminated to realize a completely field-free magnetic switch with input and output stages.

### Completely field free, reversible device operation

As mentioned before, the IMA magnet switching in our device design can be achieved by various means such as the GSHE^11^, magnetoelectric (ME) effect^12,13^, etc. In this work, we use Oersted field generated by a current pulse to switch the IMA magnet. A 50 nm thick and 6 um wide Au electrode is formed on top of the IMA magnet, isolated by 50 nm SiO_2_. This extra SiO_2_ isolation is not required, but is made for the ease of fabrication. When a current pulse is passed through the Au bar, transverse Oersted field (in “+x” direction) generated is sufficient to overcome the coercive field of the IMA magnet and switch its magnetization, thus enabling a completely field-free operation of the device. We would like to emphasize here that using Oersted field for IMA magnet switching is only for the ease of device fabrication (easy lithographic lift off process and less critical requirement of a clean interface with the IMA magnet for Au electrode compared to Ta or W) in our experiment. The well-established GSHE can be implemented to switch the IMA magnet by defining a Ta or W electrode through an etching process or a careful metallization process after *in situ* cleaning of the surface of the IMA magnet. Future generations of device designs can also utilize magnetoelectric switching or other mechanisms to further improve energy efficiency and scalability. The main focus of this work is to demonstrate that symmetry breaking dipolar field can be used to realize a spintronic logic device that contains electrically isolated WRITE and READ unit with logic information stored in the form of magnetization being transferred only from the input to the output.

**Figure 4:**
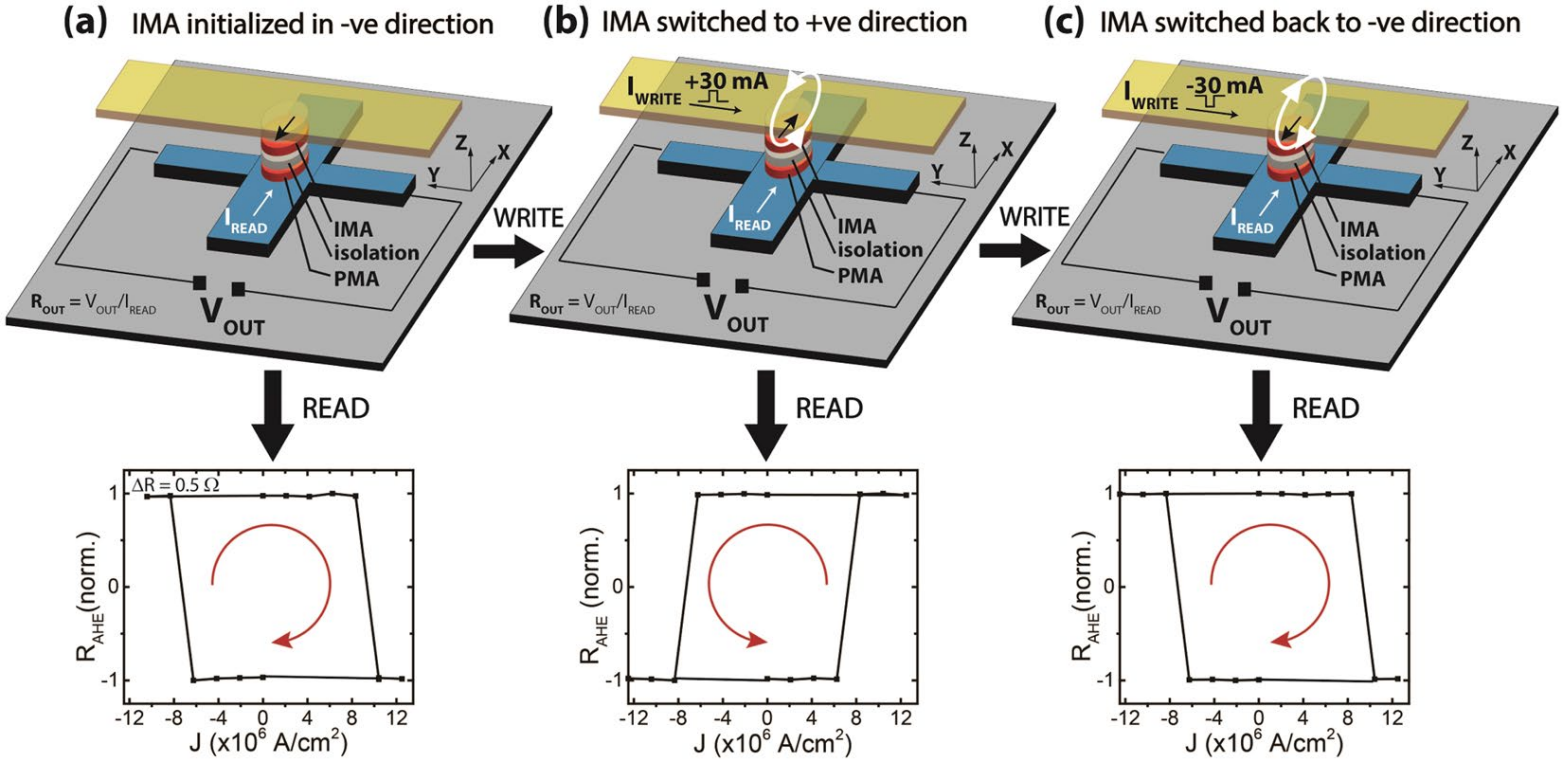
Full device operation with an Oersted field driven IMA magnet and GSHE driven PMA magnet. (**a**) The IMA magnet is initialized in the negative direction, indicated by the arrow pointing in −x direction. This is revealed by the clockwise GSHE switching loop of the PMA magnet, which serves as the READ unit. (**b**) A positive current pulse of 30 mA through the Au line generates an Oersted field that switches the magnetization of the IMA magnet to positive (WRITE operation). This is revealed by the counter-clockwise GSHE switching loop of the PMA magnet. (**c**) A negative 30 mA pulse reverses the IMA magnet direction back to negative, as revealed by the clockwise GSHE switching loop of the PMA magnet. This set of operations is completely field free and achieved by two units (IMA and PMA magnets) that are electrically isolated. Also, the information about the IMA magnet direction influences the state of the PMA magnet switching and not vice versa, hence realizing a directional coupling.

Now we demonstrate the full operation of the device in Fig. 4. First, the IMA magnet (WRITE unit) is initialized along the “−x” direction. This exerts a positive dipolar field on the PMA magnet and the AHE of the PMA magnet shows a clockwise loop. A current pulse of 30 mA is applied to the Au electrode, which switches the IMA magnet to the “+x”. This information can be read from the AHE loop of the PMA magnet, which is now in the anti-clockwise direction. Hence, the information stored in the WRITE unit, i.e., the magnetization direction of the IMA magnet, can be read from the switching direction of the PMA magnet, which serves as the READ unit. Similarly, when we apply a −30 mA pulse to the WRITE unit, it switches the IMA magnet back to the “−x” direction, and results in the clockwise AHE loop of the PMA magnet. It should be noted that reading the AHE loop direction can be done easily by applying a read current larger than the critical current and reading the sign of the output voltage. For clockwise AHE loop, we always get a negative output voltage (V_OUT_ = R_AHE_*I_READ_) and for an anti-clockwise AHE loop, we always end up with a positive output voltage.

To ensure that the GSHE switching of PMA is the responsible mechanism for the READ unit, unwanted Oersted field and Joule heating are two important mechanisms to be excluded. For our device, the Oersted field produced by the read current density of ~1 × 10^7^ A/cm^2^ through the GSHE Ta layer can be estimated by the analytical expression^24^: H_Oersted_ = μ_0_ × J × (t_GSHE_/2). Given all device dimensions, it is calculated to be about 0.5 mT and can be further reduced by scaling the thickness of the GSHE layer. In any case, this field points in the “y” direction, perpendicular to the direction of the current, and hence does not impact the switching symmetry of the device. Also, the Joule heating for a read current density of ~1 × 10^7^ A/cm^2^ is estimated to be similar to other SOT based switching schemes, owing to the similar current levels. It should be noted that in spin based logic devices, the internal energy dissipated in the magnet during switching is minimal^25^ and hence Joule heating is the major source of energy dissipation.

### Operating the device as a True Random Number Generator

**Figure 5:**
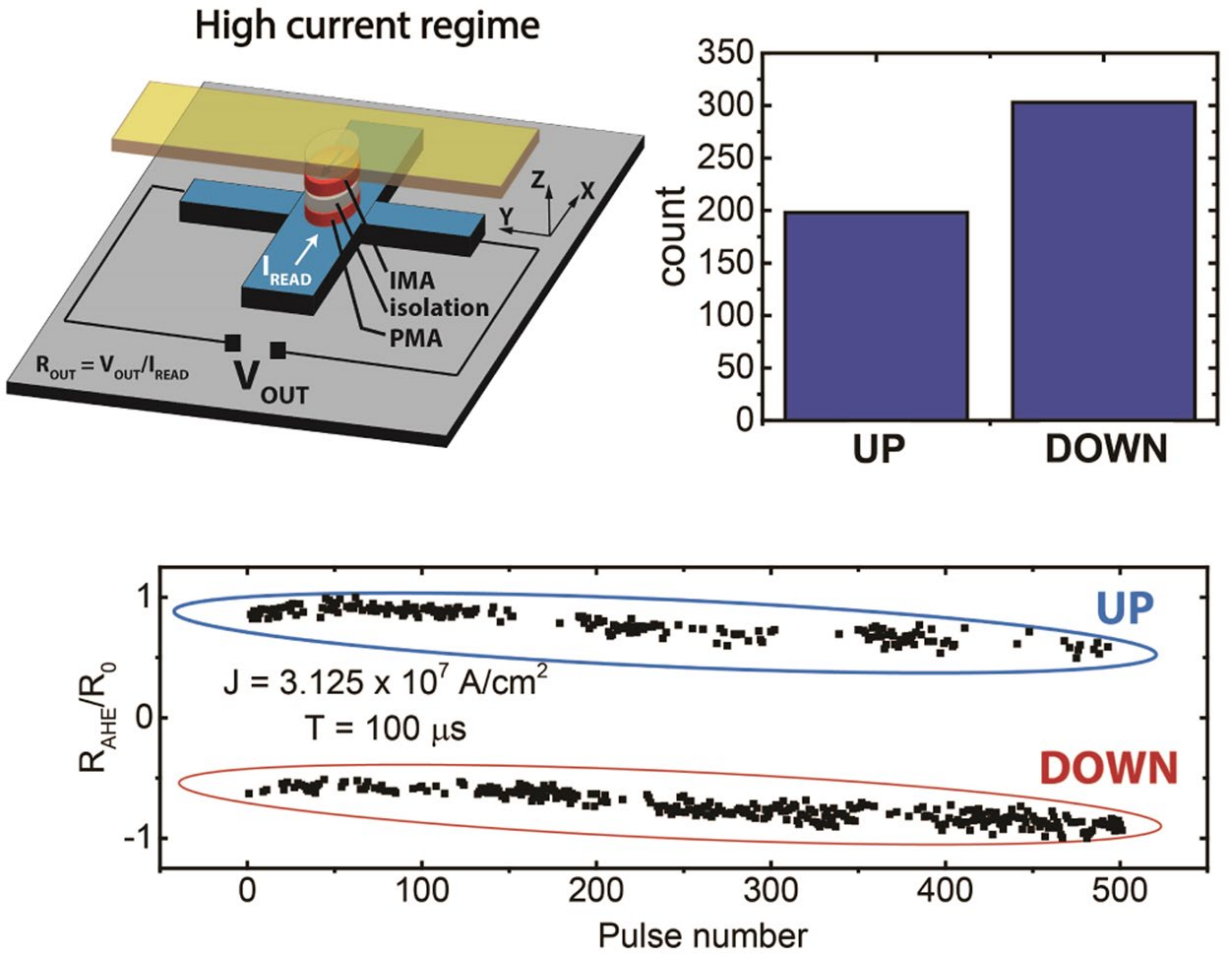
Experimentally observed stochastic switching behavior of the PMA magnet at high read currents. This is due to the PMA magnetization getting pinned along the spin polarization direction and making a stochastic choice between “up” and “down” once the current pulse is removed.

When a read current pulse with a large magnitude is applied to the Ta Hall bar, the spin torque is very high and the PMA magnet switching cannot be explained in the same way as in Section II A. Instead, the magnetization is pinned to the polarization direction of the spin current and the weak in plane field produced by the IMA magnet does not act in the same way to break the symmetry^17^. Interestingly, when the current pulse is removed, the magnetization of the PMA magnet makes a stochastic choice of arriving at either “up” or “down” direction. To achieve this, we started with a current pulse at a current density (J) slightly larger than the critical density required for deterministic switching (~1 × 10^7^ A/cm^2^ as in Fig. 4), where the magnet always remained in the state consistent with the switching loop of Fig. 4. As J just started to increase, the magnet occasionally showed random switching to the other state. When the applied J was >3 × 10^7^ A/cm^2^, the magnet state showed random choices between “up” and “down” states. As shown in Fig. 5, among 501 switching events, 303 ended at the “up” state while 198 arrived at the “down” state, showing the stochastic nature of the switching. A similar experiment was performed by Bhowmik *et al*.^26^ in a single GSHE/PMA stack. Since our device has an additional IMA magnet layer on top, it is important to note here, that the magnitude of the in plane dipolar field from the IMA magnet needs to be within a specific range in order to observe the two described operation regimes in the same device. It needs to be small enough to enable the stochastic regime under high current densities, while at the same time be strong enough to allow robust deterministic switching at low currents (details in Supplementary Information, Section S8). This optimization is much easier with a dipolar coupled in plane magnet, compared to other means of achieving field free switching.

### Circuit implications of the device

#### Functionality as a NOT or COPY gate

The operation of the device above critical currents can be summed up in the following tabular form.

From the table above, it is seen that the device operates in two regimes based on the magnitude of read current. In the deterministic logic mode, it acts as a COPY gate, i.e., V_OUT_ copies I_WRITE_ irrespective of the read current polarity. Since the output of the device is the anomalous Hall voltage, which is bipolar, this device can be turned into a NOT gate by simply reversing the output terminals, which can be useful from a circuit design perspective. Since magnetization switching in response to spin current is a thresholded operation involving a critical current, the presented device is highly non-linear. The magnetization of the IMA magnet determines the switching loop of the PMA magnet through the symmetry breaking dipolar field and hence determines the polarity of the output voltage. However, the state of PMA magnet does not affect the switching phenomenon of the IMA magnet. Hence, information only flows in one direction, i.e., from state of IMA magnet to the state of the PMA magnet, ensuring directionality. This device has a built in memory as the information is stored in the state of a nanomagnet, which is non-volatile and can be stable for several years by proper design.

#### Operating power

Any CSL type of spin logic devices, where information gets transferred from the input to the output by coherent switching of two isolated nanomagnets, will always require energy to switch both of the nanomagnets, albeit it is through GSHE switching of one magnet and dipole coupling to the other in the original CSL proposal^2^. In ref.^2^, this was manifested in the required switching current being 2*I_sw_ (I_sw_ being the switching current for one of the magnets). In our device, the energy is partially divided to the write operation and partially to the read operation, instead of the “traditional” way, where most energy is spent in the write operation and a much smaller energy is spent in the read operation. The write operation can be made ultra-low power by use of magnetoelectric (ME) effect for switching the IMA magnet of the WRITE unit. The read operation involves SOT switching of the PMA magnet of the READ unit, which requires less power compared to the SOT or STT switching of IMA magnets that are used in current MRAM technology (see Supplementary Information, Section S10). Hence, the total operation energy (read plus write) can be significantly smaller than many of the proposed CSL like spintronic logic devices^2,27,28^.

Note that the operation of this spin logic device is different from that of a CMOS logic device. Owing to its non-volatility, the V_DD_ power supply can be a clock instead of being constantly present. Therefore, there is no constant energy loss through the V_DD_-ground path.

#### Concatenability

If the AHE output is driving a capacitive load, (e.g. the magnetoelectric WRITE unit of the next stage) then the required V_DD_ to drive the next stage is given by:

2 $${V}_{DD}={V}_{SW,IMA}\times \frac{{\rho }_{0}}{{\rho }_{AHE}}\times \frac{{L}_{PMA}}{{W}_{PMA}}\times (1+\frac{{t}_{GSHE}}{{t}_{PMA}})$$

where V_SW,IMA_ is the critical voltage for switching the IMA magnet of the next stage. By proper geometrical design, the last two factors of the above equation can be reduced to 1/3, whereas for a standard material like CoFeB, the factor ρ_0_/ρ_AHE_ is ~30 (ref.^29^). Hence, the required V_DD_ would be ≈10 × V_SW,IMA_. The recent advances in voltage driven 180 degree magnetization switching^12,13^ can lead to an ultra-low V_SW,IMA_, enabling a low V_DD_ operation of this device. Details of the analysis and a schematic of envisioned circuit connection is given in Supplementary Information, Section S9. Fan-out can be improved by replacing the AHE reading scheme by a PMA MTJ, where the free layer of the MTJ receives symmetry breaking field from the IMA magnet. Using MTJ resistance instead of AHE comes at the cost of compromising the bipolar nature of output.

#### Stochastic mode

In the stochastic mode of operation, this device gives the unique functionality of true random number generation that is not easily feasible with CMOS elements. Having a hardware random number generator made of the same device in a logic chip can be very useful for applications such as cryptographic systems^30^.

## Conclusion

We have demonstrated an experimental implementation of a spin logic device with the essential properties of (i) WRITE unit, (ii) READ unit, (iii) directional coupling, and (iv) electrical isolation. This is achieved by combining an IMA and PMA nanomagnet in a vertical stack, utilizing the dipolar field for information transfer. The device has high non-linearity, directionality as well as inbuilt memory. The device can be used as a COPY gate or a NOT gate, by simply choosing the polarity of output terminal connections. The electrical write and read mechanisms can be easily replaced by established technology such as GSHE or emerging technology such as ME for write, and MTJ for read to reduce energy consumption and make the future generation of this device more efficient. Finally, this device has a stochastic regime of operation under high current pulses, which serves as a hardware form of a true random number generator, desirable in many computing applications.

## Methods

### Sample Preparation and characterization

The composite IMA-PMA stack was prepared on a thermally oxidized silicon substrate by PVD magnetron sputtering. Along with the main sample, one control sample was placed in the chamber. After depositing till the MgO isolation layer, the vacuum was broken and the first control sample was taken out. Another control sample was placed in the chamber along with the main sample and the deposition of the IMA layer was carried out. It is worth mentioning that the Ta layer inserted between the two MgO layers in Fig. 2(a) is not necessary for the device. It was inserted to be consistent with a previously developed recipe for PMA film deposition. m-H loop characterization of all samples were done in Quantum Design MPMS-3.

### Device Fabrication

The device was made in a four step e-beam lithography process. First, the entire stack was etched into the Hall bar shape by e-beam lithography and dry etching using Argon plasma, followed by removal of the HSQ etch mask. Then, the elliptical island containing the IMA magnet, PMA magnet and the isolation layers was formed on top of the Hall bar by e-beam lithography and Ar plasma etching, where the etching was stopped just before the bottom Ta layer. The top Au bar for providing Oersted field was formed by a third e-beam lithography step, followed by e-beam evaporation of SiO_2_(50 nm)/Au(50 nm) and liftoff in acetone. Finally, the contact pads were formed on the Hall bar by another e-beam lithography step followed by e-beam evaporation of Ti(20 nm)/Au(100 nm) and liftoff in acetone. After the device fabrication, an annealing step was carried out at 250 °C for one hour in a vacuum chamber with ~3 × 10^−8^ Torr pressure in the absence of any magnetic field. Also, reference stacks for independent magnetic characterization of the IMA and the PMA magnet layers were annealed at the same time, before measuring the magnetic hysteresis loops of Fig. 2(b,c).

### Measurement setup

The anomalous Hall effect measurement were done using a sinusoidal current from a Keithley 6221 current source and a SRS 850 DSP lock-in amplifier. The same Keithley 6221 was also used to generate current pulses for SOT switching of the PMA magnet and Oersted field switching of the IMA magnet. All measurements were carried out in a Lakeshore probestation, which also provided the required in-plane field for the measurements in Fig. 3(b). Anomalous Hall effect measurement with out of plane magnetic field were done in Quantum Design PPMS Dynacool.

### Data availability

The data that support the plots within this paper and other findings of this study are available from the corresponding author upon reasonable request (Table 1).

**Table 1:** Deterministic and stochastic operation of the presented device.

Operation	I_WRITE_	I_READ_	R_AHE_	V_OUT_ (=I_READ_ × R_AHE_)
Deterministic Logic Gate (Moderate I_READ_)	+	+	+	+
	+	−	−	+
	−	+	−	−
	−	−	+	−
TRNG (Large I_READ_)	N.A.	+/−	random	random

## Electronic supplementary material

Supplementary Information
