# Supplementary material for: Experimental Demonstration of a Spin Logic Device with Deterministic and Stochastic Mode of Operation
Source: Sci Rep. 2018 Jul 30;8:11405. doi: 10.1038/s41598-018-29601-5 (PMC6065380; doi:10.1038/s41598-018-29601-5)
Supplement: Supplementary file 1 — Supplementary Information [file 41598_2018_29601_MOESM1_ESM.pdf]

Experimental Demonstration of a Spin Logic Device with Deterministic and Stochastic Mode of Operation

Punyashloka Debashis[1,2] and Zhihong Chen[1,2]
[1]School of Electrical and Computer Engineering, [2]Birck Nanotechnology Center
Purdue University, West Lafayette, IN 47907, USA

SUPPLEMENTARY MATERIAL

Contents:

S1: Field free switching of another nominally identical device

S2: Switching phase plot

S3: Deterministic switching- effect of current polarity

S4: Excluding the possibility of domain wall propagation to explain our field free switching

S5: Confirming zero field of the probe station

S6: Contribution of IMA magnet and PMA magnet to the AHE signal

S7: MgO isolation layer leakage characterization

S8: Choosing isolation layer thickness for enabling stochastic regime

S9: Integrating AHE READ and magnetoelectric WRITE for future generations

S10: Critical current for SOT switching of IMA magnet and PMA magnet

S1: Field free switching measurement of another nominally identical device:

The field free switching measurements on another nominally identical device is shown in Figure S1. First we measure the switching loops in the presence of an external field, this time at a smaller magnitude of around 10 mT (Figure S1 (a)). The loop direction reverses with the external field direction. Then we carry out field free switching by initializing the IMA first in the positive direction, followed by switching loop of the PMA. The loop direction corresponds to the negative B_{ext}. Then we initialize the IMA to the negative direction and see that the PMA switching loop direction reverses. We repeat this process 4 times, to see that the PMA's switching loop always changes with the IMA direction, confirming robust field free switching (Figure S1 (b)).

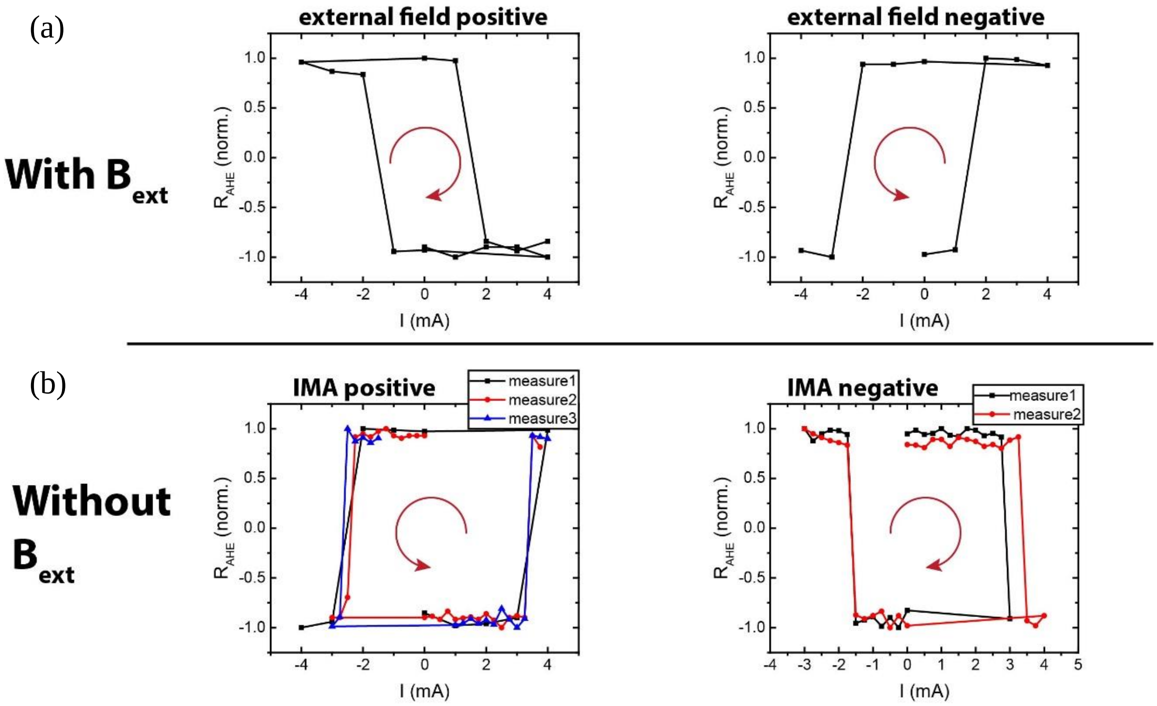

Fig S1: Field free switching of another nominally identical device. The field free switching loops are repeated to show the robust operation.

S2: Switching phase plot:

To verify the mechanism of PMA switching with in plane polarized spin current, we carried out external field assisted switching on another device with varying field. The critical current required to switch the magnetization reduces with increasing external field, as shown in the measured switching phase diagram (Fig. S2). Our experimentally measured phase plot has similar shape and symmetry that predicted by Liu et al.[17].

The crosses represent the cases of field free switching.

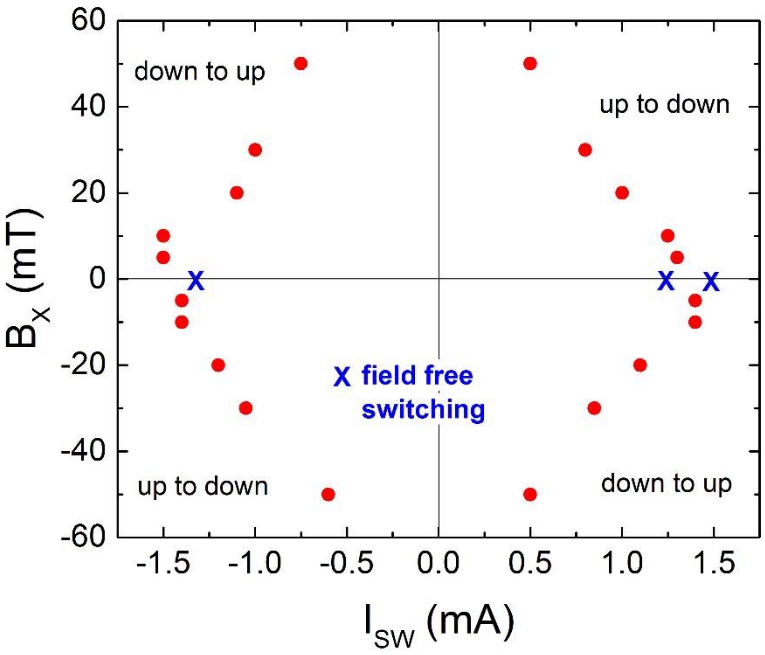

Fig S2: Phase diagram showing critical switching current vs. applied external field for another device. The crosses are for the zero external field switching cases.

S3: Deterministic switching- effect of current polarity:

Further test is carried out to ensure the robustness of the field free deterministic switching. To ensure that the switching is dependent on the current polarity and not just on the magnitude, we passed a series of pulses in random order of positive and negative polarity. As seen in Fig S3 (a), the magnetic state changed according to the current polarity, confirming that it was due to the SOT of GSHE current in the presence of the dipolar field from the IMA. We also, applied pulses of increasing magnitude and observed the Anomalous Hall resistance changing abruptly only when a critical current is reached (Fig S3 (b)).

(a)

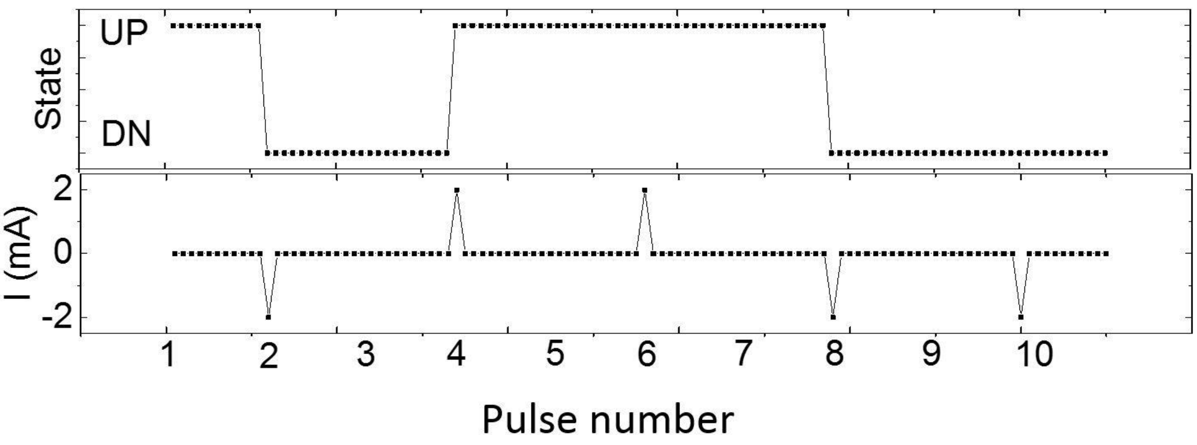

(b)

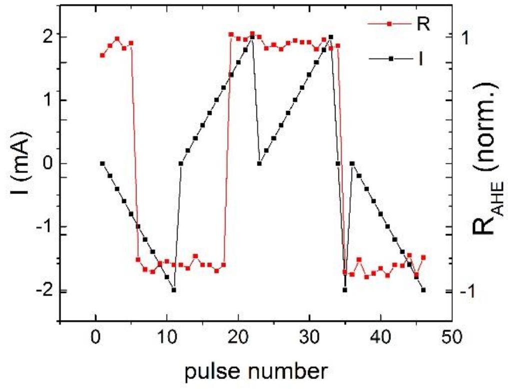

Fig S3: Magnetization switching shows dependence on current polarity, excluding heating effect or stochastic switching. This further confirms the robust deterministic switching under moderate read currents.

S4: Excluding the possibility of domain wall propagation to explain our field free switching:

We exclude the possibility of domain wall propagation being the cause of the field free switching in our device. In reference 22 of the main text, field free switching is obtained by initiating domain wall nucleation, followed by propagation, depending on the initial direction of the PMA magnet. That is to say that the PMA switching loop is always anti-clockwise if the PMA is initialized in the UP direction, while it is always clockwise if the PMA loop is initialized in the DOWN direction in reference 22, Figure 3). On the contrary, we show that we can obtain both clockwise and anti-clockwise switching loop directions, starting from $M_{initial}$ to be DOWN. In our case, the loop direction is determined by the IMA direction but not the PMA's initial condition, as shown in Figure S4.

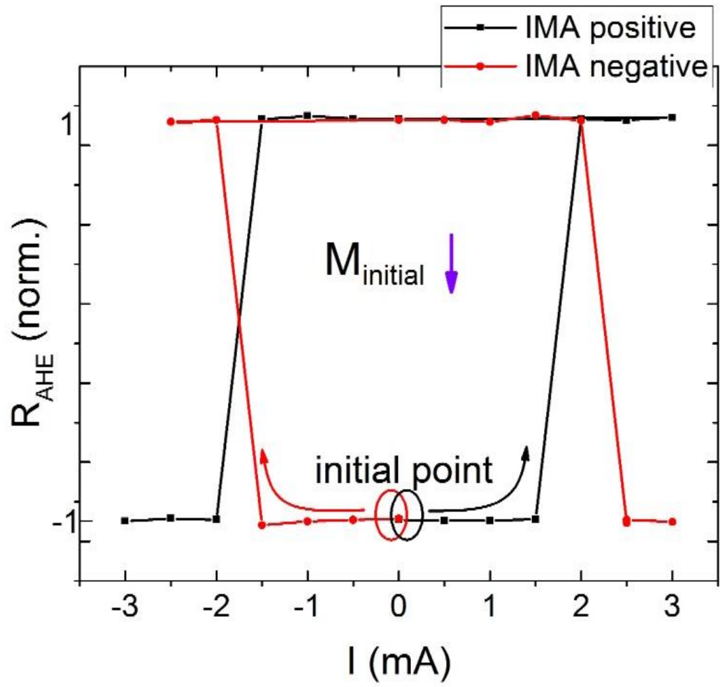

Fig S4: Starting from PMA magnetization in the DOWN direction (-Z direction), we obtain a clockwise switching loop (red curve) for the IMA initialized in -X direction and anti-clockwise switching loop (black curve) for the IMA initialized in +X direction. Since the loop direction reverses even for the same starting M_Z, we can exclude the possibility of switching by the reverse domain wall nucleation/propagation phenomenon seen in reference 22.

S5: Confirming zero field in the probe station:

The Lakeshore probe station used to carry out the measurements had coils and a field concentrator to provide in plane magnetic fields for measurements. It is equipped with a Gaussmeter that reads the magnetic field in real time. For field free switching experiments we wanted to make sure that the Gauss meter reads zero field only when the field near the sample is actually zero. For this purpose, we carefully followed the Gaussmeter nulling protocol using a mu-metal shield provided by Lakeshore. However, to further ensure that the field is zero at the sample position, a second harmonic AHE measurement of a PMA device in presence of in plane field was taken at the exact same position where the IMA-PMA device is measured. Ideally, the second harmonic AHE signal should be zero for zero field and should be shifted if there is any additional stray field, when the gauss meter reads zero. From Fig S3, we see that the AHE signal goes to zero for zero field read by the Gaussmeter (x-axis), confirming that there is no significant stray field from any sources.

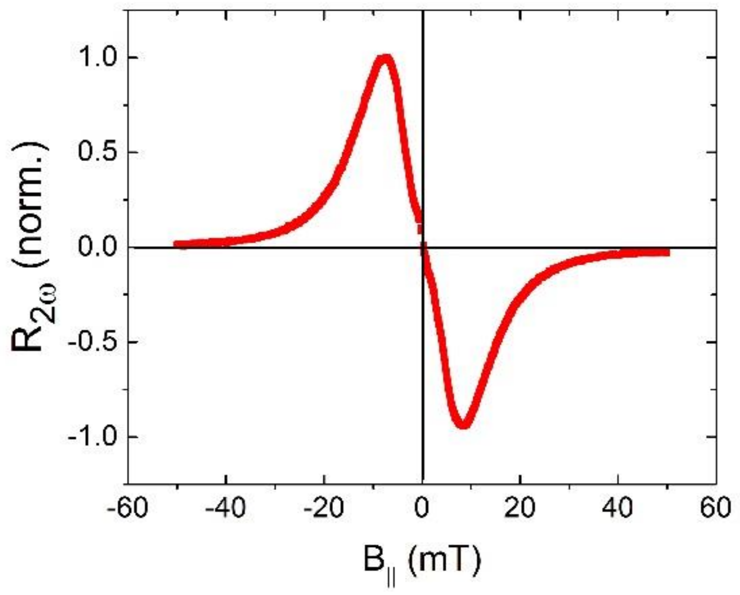

Fig S5: AHE loop crossing (0, 0) in this confirms the zero field condition in the probe station.

Please note that the resistance curve had a vertical offset due to lithographic misalignment of the voltage and current arms of the hall bar. This offset was subtracted by looking at the two saturation values at +/-50 mT. The saturation value should also be zero and hence was used as a reference to cancel the offset.

S6: Contribution of IMA and PMA to the AHE signal:

To make sure that the signal we measure from the AHE does not have any contribution from the IMA magnet, we compare the electrical measurement of a fabricated device with the m-H measurement of the entire stack by Quantum Design MPMS-3.

First, from the m-H measurement of the entire stack under out of plane field (Fig. S5 (a)), we observe abrupt changes in magnetization in the low field regime, which is a result of the switching of the PMA. However, at high fields, a gradually saturating curve is measured, which is reminiscent of the out of plane hard axis saturation of the IMA magnet. A device fabricated with common contacts to the PMA and IMA magnet shows the same behavior for R_{AHE} (Fig. S5 (b)). Here, the common contacts makes the current to flow in both of the magnets and hence the Hall voltage read-out has contributions from both magnets.

Now we make the same measurement on our final device, which has an etched magnet island on top of the bottom Hall bar. In this case the bottom Hall bar only contacts the bottom PMA magnet and the measured Hall voltage does not include any contribution from the IMA magnet as it is isolated by the MgO layer. This is evident as the saturating feature is missing from the R_{AHE} loop at high fields (Fig. S5 (c)).

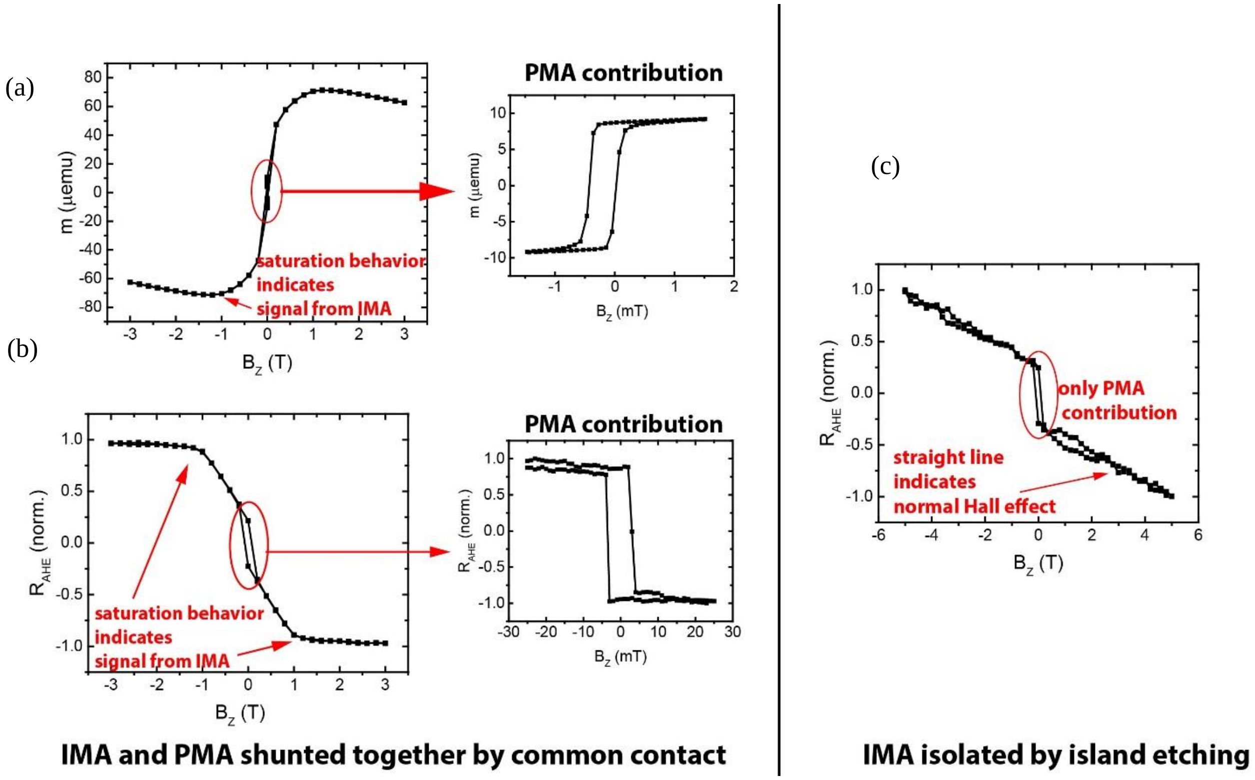

Fig S6: Contribution to Hall voltage. (a) SQUID VSM measurement of the composite IMA-PMA stack. (b)AHE resistance of a device with contacts made to both of the IMA and PMA magnets. (c) AHE resistance of a device where the magnet stack is etched to form an island on top of the Hall bar. In this device, the IMA is isolated from the PMA and the Hall bar by the MgO layer. Hence it does not contribute to the measured AHE signal.

S7: Isolation layer leakage characterization:

To ensure proper isolation between the IMA and PMA magnet, we used a 6-7 nm MgO layer. To characterize the leakage of this layer, we deposited a stack shown in Fig S4 (a) and then fabricated a device shown in Fig. S4 (b). In this device, the cross-section of the current flow is 20 um X 20 um, defined by the area of the intersection between the top Gold contact and the bottom patterned stack, with a 60-70 nm thick SiO_2 layer isolation. The leakage was characterized by an Agilent parameter analyzer. Fig. S4 (c) and (d) show the resistance as a function of applied voltage across the MgO layer for two nominally identical devices. It is clear that the resistance is 10 Tera-Ohms to few hundred Mega-Ohms for the range of applied voltages. An important point to note here is that in the IMA-PMA device, we apply a current of ~2 mA across a device with resistance of around 2 KΩ, resulting in ~ 4 V voltage drop across the length of the current leg. Hence, the maximum voltage drop that the MgO isolation layer has to withstand is less than 4 V.

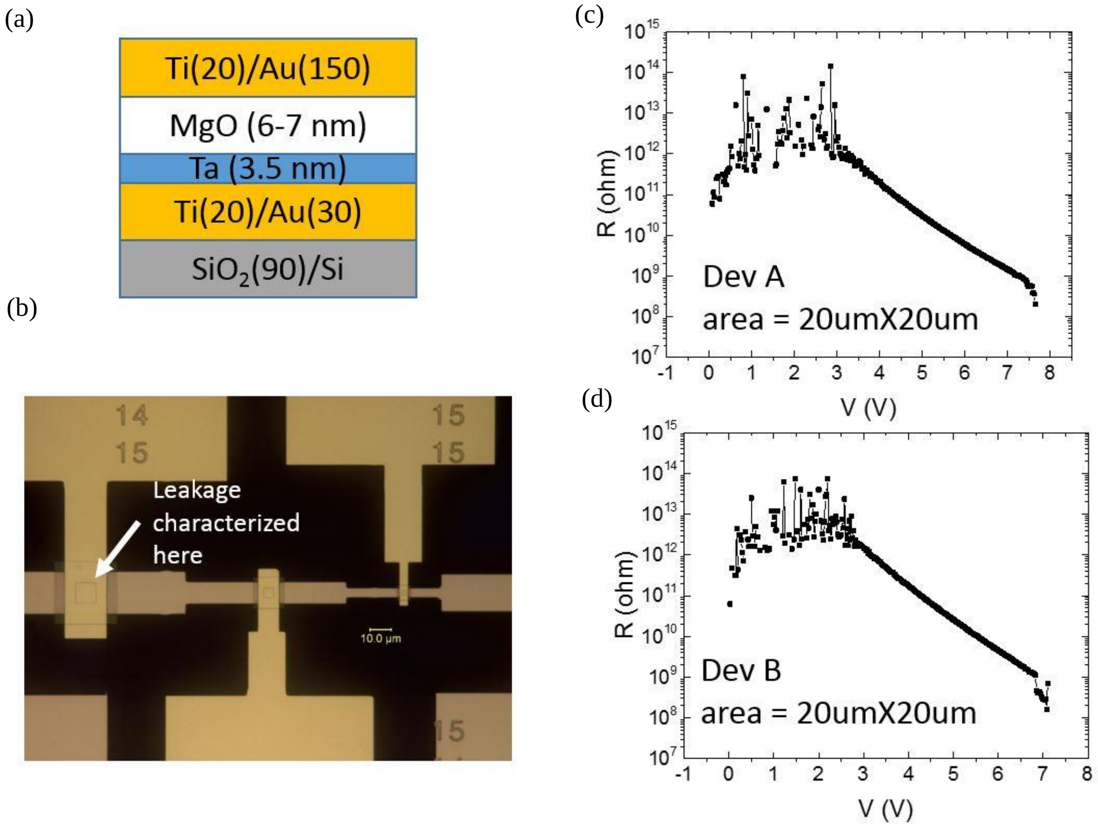

Fig S7: Leakage characterization of the MgO isolation layer. (a) Stack used for leakage characterization. (b) Optical image of the fabricated device. (c), (d) Resistance across the MgO isolation layer as a function of applied voltage. The MgO layer provides excellent isolation and starts to show signs of breakdown around 7.5 V, which corresponds to ~1V/nm..

S8: Isolation layer thickness choice for stochastic operation regime:

The deterministic switching regime in our device can be achieved by GSHE current that generates moderate spin torque. As explained by Liu et al.[17], in this regime, the magnetization remains in the z-x plane and an in plane symmetry breaking field along x direction is required in the PMA magnet response to the GSHE spin torque. Hence, B_X can be as small as needed for robust operation. In our device, B_X is a dipolar field of around 1mT from the IMA magnet and is sufficient to determine the switching direction of the PMA magnet in presence of the GHSE spin torque.

However, in the high current regime, the spin torque is large. Accordingly, the magnetization vector develops a component along the Y direction (according to our convention defined in all figures in the main text), and the equilibrium magnetization vector is pinned almost in the X-Y plane, with a small "Z" component, given by[17]:

$$m_Z = -B_X/ \tau_{ST}^0$$

Therefore, with large current densities corresponding to sufficiently strong spin torque (τ_{ST}^0) and sufficiently small external fields (B_X), m_Z is close to zero, i.e, the magnetization is pinned to the X-Y plane. In this case, once the spin torque is removed, the magnet makes a natural probabilistic choice to go back to either +Z or –Z direction. The PMA magnetization read from the measured AHE voltage thus outputs +V or –V randomly.

It is important that the ratio B_X/τ_{ST}^0 should be small enough in this regime to ensure stochastic behaviors. However, τ_{ST}^0 cannot be made arbitrarily large as it would require large current densities that will damage the device structure due to Joule heating. Therefore, B_X should be small enough so that m_z can be close to zero even at practical current densities. In our device, we achieve this condition at a current density of 3.1 x 10^7 A/cm^2, which is not too high to damage the device even after repeated measurements. We demonstrate that by carrying out 500 switching events.

In our device B_X is provided by the dipolar field from the IMA on top of the stack and hence is dependent on the thickness of the MgO isolation layer and the magnet dimensions. Figure S8 shows the volume averaged B_X on the PMA, numerically calculated in MATLAB. The required B_X range can be achieved by choosing the proper MgO thickness at given magnet dimensions. For the 3 um x 1 um magnet used in this work, the calculated dipolar field is marked with a circle in Figure S8.

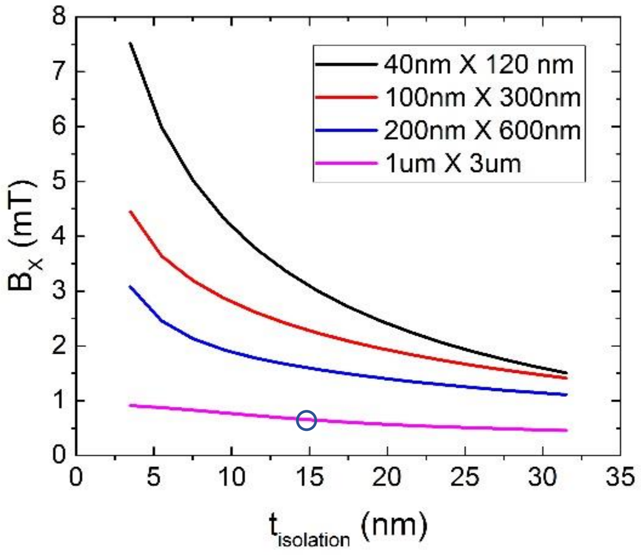

Fig S8: "X" component of the IMA dipolar field vs. MgO isolation layer thickness for various magnet dimensions. Depending on the magnet size, an isolation layer thickness should be chosen to provide enough dipolar field (B_X) for robust deterministic switching while not too large to allow stochastic behaviors at higher currents. The marked circle indicates the dimensions used in this work with the estimated dipolar field of 0.7 mT.

S9: Integrating AHE READ and voltage controlled WRITE for future generations:

Anomalous Hall effect has been used in the present device as the mechanism to read the stored information. However, a more energy efficient method such as voltage controlled magnetoelectric effect (ME) to switch the IMA magnet is preferred. The envisioned future device and its possible concatenation with an integrated ME WRITE unit is shown in the diagram below.

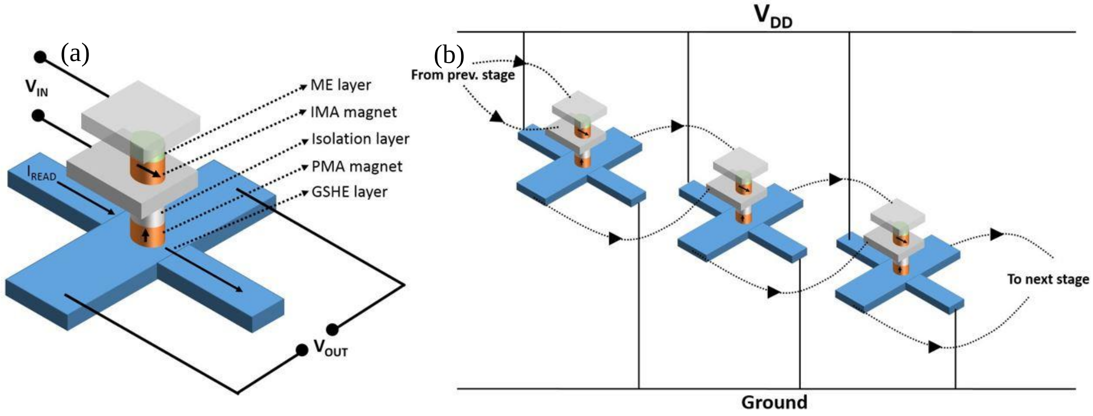

Fig S9: (a) Individual device with ME WRITE and AHE READ. (b) Concatenation scheme. The V_{DD}-Ground line through the GSHE layer provides the I_{READ}. Note that due to the non-volatility of the device, the V_{DD} need not be present continuously, leading to no static power loss different from the CMOS.

Here, the V_{OUT} (=V_{AHE}) will be applied across an effective capacitor and hence form a DC open circuit. In this case, the analysis goes as follows (including I_{READ} shunting consideration through PMA magnet and GSHE layer):

$$V_{OUT} = V_{AHE} = (\rho_{AHE}/t_{PMA})*I_{READ} = (\rho_{AHE}/\rho_0)*(W_{PMA}/L_{PMA})*V_{DD}*[1+ (t_{GSHE}/t_{PMA})]$$

This V_{OUT} is the critical switching voltage for the IMA magnet of the next stage. Therefore:

$$V_{OUT} = V_{SW,IMA}$$

⇨ $V_{DD} = V_{SW,IMA}*(\rho_0/\rho_{AHE})*(L_{PMA}/W_{PMA}) *[1+ (t_{GSHE}/t_{PMA})]$

Practically, a ratio of $(L_{PMA}/W_{PMA}) = 1/6$ can be achieved by design.
A ratio of $(t_{GSHE}/t_{PMA}) \approx 1$ can be achieved as $t_{GSHE} \approx 1$ nm has been experimentally demonstrated[SR1].
Therefore, the last two factors can be reduced to ≈ 1/3
For a standard material like CoFeB, $(\rho_0/ \rho_{AHE}) \approx 30$ (reference 29) and can be as low as 25 for Heusler alloys[SR2] and 5 for Weyl semimetals[SR3].
Therefore, the following can be achieved practically by using currently available technology and material systems like CoFeB: $V_{DD} \approx 10*V_{SW,IMA}$
For voltage driven 180 degree switching of IMA magnet, the experimentally demonstrated value of required electric field is in the range of 40 V/um for BFO/CoFe stacks[12], and ~ 1 V/um for strain mediated magnetization reversal using PMN-PT/CoFeO stacks[13]. Therefore, with proper scaling of the ME material thickness, $V_{SW,IMA}$ of ~10 mV is feasible, leading to a V_{DD} of ~100 mV, which is a reasonably small voltage read-out requirement.

S10: Critical current for SOT switching of IMA and PMA:

The expressions of the critical current for SOT switching of 40 k_BT IMA and PMA magnets are given by[17]:

$I_{sw,IMA} = \frac{4e}{\hbar}\alpha(40k_BT)(1+\frac{H_D}{2H_K})(\frac{t_{GSHE}+(\sigma_{IMA}/\sigma_{GSHE})t_{IMA}}{L_{IMA}})(\frac{1}{\theta_{SH}})$ for the IMA magnet case, and

$I_{sw,PMA} = \frac{2e}{\hbar}(40k_BT)(\frac{t_{GSHE}+(\sigma_{PMA}/\sigma_{GSHE})t_{PMA}}{L_{PMA}})(\frac{1}{\theta_{SH}})$ for PMA magnet switching at an optimized symmetry breaking in plane field.

Hence, the required current to switch a PMA magnet is less than that for an IMA magnet with the same energy barrier by a factor of

$$\frac{I_{sw,IMA}}{I_{sw,PMA}} \approx 2\alpha\left(1+\frac{H_D}{2H_K}\right)\left(\frac{t_{GSHE}+t_{IMA}}{t_{GSHE}+t_{PMA}}\right)$$

(Here we have considered $\sigma_{IMA} \approx \sigma_{PMA} \approx \sigma_{GSHE}$, which is reasonable for CoFeB/Ta stacks)

Considering values of $\alpha = 0.015$, $H_D/H_K = 150$, $t_{GSHE} = 1$ nm, $t_{IMA} = 1$ nm and $t_{PMA} = 2$ nm, the switching current for a PMA magnet is >3 times smaller than what is needed for an IMA magnet. Consequently, less power is required for the READ function of our device compared to the write power consumption by SOTMRAM, which is already touted to be better than STTMRAM.
Note that, for the switching current comparison between IMA and PMA magnets, we have ignored heating effects that are taken into account in the expression given in reference 17.

REFERENCE

SR1: Kim, J. et al. Layer thickness dependence of the current-induced effective field vector in Ta| CoFeB| MgO, Nat. Mater. 12, 3, 240 (2013).

SR2: Fu, H.R., Ma, L., Tian, N., You, C.Y. & Wang, K. The enhancement of anomalous Hall effect by inserting MgO layer in perpendicular anisotropic Pd/Co2MnSi/MgO/Pd films, AIP Adv. 8, 5, 055804 (2018).

SR3: Liu, E. et al. Giant anomalous Hall angle in a half-metallic magnetic Weyl semimetal. Preprint at: https://arxiv.org/abs/1712.06722v2 (2017).
